# Catch me if you can: a biotinylated proteoliposome affinity assay for the investigation of assembly of the MexA-MexB-OprM efflux pump from *Pseudomonas aeruginosa*

**DOI:** 10.3389/fmicb.2015.00541

**Published:** 2015-06-02

**Authors:** Véronique Yvette Ntsogo Enguéné, Alice Verchère, Gilles Phan, Isabelle Broutin, Martin Picard

**Affiliations:** Laboratoire de Cristallographie et RMN Biologiques, Faculté de Pharmacie de Paris, UMR 8015 CNRS – Université Paris 089 Descartes, Paris, France

**Keywords:** multidrug resistance, efflux pump, membrane protein, proteoliposome, macro-molecular assembly

## Abstract

Efflux pumps are membrane transporters that actively extrude various substrates, leading to multidrug resistance (MDR). In this study, we have designed a new test that allows investigating the assembly of the MexA-MexB-OprM efflux pump from the Gram negative bacteria *Pseudomonas aeruginosa*. The method relies on the streptavidin-mediated pull-down of OprM proteoliposomes upon interaction with MexAB proteoliposomes containing a biotin function carried by lipids. We give clear evidence for the importance of MexA in promoting and stabilizing the assembly of the MexAB-OprM complex. In addition, we have investigated the effect of the role of the lipid anchor of MexA as well as the role of the proton motive force on the assembly and disassembly of the efflux pump. The assay presented here allows for an accurate investigation of the assembly with only tens of microgram of protein and could be adapted to 96 wells plates. Hence, this work provides a basis for the medium-high screening of efflux pump inhibitors (EPIs).

## Introduction

In Gram-negative bacteria, efflux transporters play a major role in the emergence of antibiotic resistance thanks to their ability to export drugs out of the cell (Nikaido, 2009). They are organized as tripartite systems where the RND pump (member of the Resistance, Nodulation, cell Division family) is located in the inner membrane and works in conjunction with a periplasmic protein belonging to the MFP family (Membrane Fusion Protein family), and an outer membrane channel (from the OMF, outer membrane factor family). The cytoplasmic inner membrane protein acts as an energy-dependent pump with broad substrate specificity. The outer membrane protein acts as a porin whereas the third one is thought to stabilize the whole complex. The understanding of these pumps has been largely improved thanks to the publication of high-resolution structures of the various components (Hinchliffe et al., 2013) but the overall mechanism of assembly as well as the stoichiometry of assembly is still a matter of controversy. Two main models have been suggested for a complete description of the pump assembly. In the first one there is a direct docking of the RND protein on the OMF. In this model, three or six copies of the MFP stabilize the complex. This direct docking model has been comforted by the characterization of a direct interaction between AcrB and TolC by cross-linking (Tamura et al., 2005; Symmons et al., 2009; Weeks et al., 2010) and by *in vitro* surface plasmon resonance (SPR; Tikhonova et al., 2011). A second model where the MFP, assembled in a funnel-like conformation with a stoichiometry of 6- 12 copies acts as a bridge between the RND and the OMF, has been comforted by recent electron microscopy studies (Trépout et al., 2010; Du et al., 2014; Kim et al., 2015). This controversy builds up on a series of biochemical and biophysical characterizations. Indeed it is known for long that a genuine tripartite complex can be purified, without prior cross-linking, simply by using a hexahistidine tag on each of the proteins (Tikhonova and Zgurskaya, 2004). The assembly can be also studied *in vivo*, by screening mutants and assessing their ability to restore resistance against antibiotics (see, e.g., Nehme and Poole, 2007) or by taking advantage of the sensitivity of bacterial cells to vancomycin, a very large antibiotic that cannot penetrate the cell by slow passive diffusion (Bavro et al., 2008): Gram negative bacteria are not sensitive to vancomycin but become so if the OMF were to open upon assembly, thereby allowing the antibiotic to diffuse inside the cell. This strategy has been used to study the efflux pump MtrC-MtrD-MtrE (respectively MFP-RND-OMF) from *Neisseria gonorrhoea* (Janganan et al., 2013). The assembly has also been studied *in vitro* by isothermal titration calorimetry (ITC), a technique that allowed for the evaluation of the affinity between components and the establishment of a four-stage model where four sequential, non-cooperative, binding sites are suggested (Touzé et al., 2004). Following similar lines, the assembly of the whole complex has been studied by (SPR; Tikhonova et al., 2011). Upon injection of the protein partners (premixed or in a sequenced manner) on a biochip onto which AcrB was adsorbed, they could monitor assembly in real time. The tripartite complex successfully formed only when the partners were mixed in following a specific sequence of events.

In spite of the valuable and continued efforts for understanding how a tripartite efflux pump assembles, the development of original solutions compatible with medium-high screening of inhibitors is lacking. Antibiotic resistance is an increasing threat complicated by the scarcity of new anti-infective drug families under development. Hence, finding new ways to inhibit efflux pumps is a very promising and important endeavor because it was shown that such inhibitors restore the activity of antibiotics (Lomovskaya et al., 2006; Pages and Amaral, 2009). Lately, we have described a new protocol where transport through the whole pump could be monitored by use of proteoliposomes and respective fluorescent probes liable to report the activity of each partner of the pump (Verchère et al., 2015). As a complementary approach we present here a new strategy that allows investigating the formation of efflux pumps.

## Materials and Methods

1,2-dioleoyl-sn-glycero-3-phosphocholine (DOPC) and 1,2-dioleoyl-*sn*-glycero-3-phosphoethanolamine-N-(cap biotinyl; DOPE, cap biotin) were purchased from Avanti Polar Lipids (reference: 850375C and 870273C, respectively). SM_2_ Bio-beads were obtained from Bio-Rad (reference 152-3920). n-dodecyl-β-D-maltopyranoside (DDM) and n-octyl-ß-D-glucopyranoside (ß-OG) was purchased from Anatrace (reference D310LA and O311, respectively).

### Production, Purification of MexB

MexB was purified following the protocol described in (Mokhonov et al., 2005), with minor modifications. Heterologous expression was performed in a C43 ΔAcrB strain. A pre-culture was grown overnight at 37°C under agitation at 200 rpm and inoculated at OD_600nm_ = 0.05 in 2xTY medium containing 100 μg/mL of ampicillin. Cell were grown to OD_600nm_ = 0.6 at 30°C at 200 rpm and then cooled down at 4°C for 30 min. Isopropyl β-D-1- thiogalactopyranoside (IPTG) was added (1 mg/mL final) and growth was continued overnight at 20°C. Cells were then lysed thanks to a cell-disrupter (Cell-D from Constant LTD) at 4°C after two passages at 2.4 kbar. Membranes were collected upon centrifugation at 100,000 g during 1 h at 4°C. Solubilization was performed overnight at 4°C in 10 mM Bis-Tris pH 7.4; glycerol 20%, 10 mM imidazole and 500 mM NaCl at a 2:1 detergent-to-protein ratio (protein concentration was determined using the Bicinchoninic acid test from Sigma). Purification was performed by affinity chromatography followed by a gel filtration (superose 6 HR, GE).

### Production, Purification of MexA

MexA was purified following the protocol described in (Akama et al., 2004) with minor modifications. Heterologous expression was performed in a C43 ΔAcrB strain. A pre-culture was grown overnight at 37°C under agitation at 200 rpm and inoculated at OD_600nm_ = 0.05 in 2xTY medium containing 25 μg/mL of chloramphenicol. Cell were grown to OD_600nm_ = 0.6 at 30°C at 200 rpm and then cooled down at 4°C for 30 min. Arabinose was added (0.02% final, w/v) and growth was continued during 2.5 h at 30°C. Cells were then lysed thanks to a cell-disrupter (Cell-D from Constant LTD) at 4°C after two passages at 2.4 kbar. Membranes were collected upon centrifugation at 100,000 g during 1 h at 4°C. Solubilization was performed overnight at room temperature in 20 mM Tris-HCl pH 8, glycerol 10%, 15 mM imidazole at a 40:1 detergent-to-protein ratio (protein concentration was determined using the Bicinchoninic acid test from Sigma). Purification was performed by affinity chromatography followed by a gel filtration (superose 6 HR, GE). A truncated form of MexA “cysteine-less,” named MexAnp, without its first 24 residues, has been generated in the laboratory in order to produce and purify a non-palmitoylated, soluble version of the protein.

### Production, Purification of OprM

OprM is purified following the protocol described in (Phan et al., 2010) with minor modifications. In brief, the procedure was exactly that described above for MexA except that an additional solubilization step is added on the membrane pellet: N-octylpolyoxyethylene (C_8_POE) is added (2% final, w/v) and the mixture was incubated 30 min at 37°C. C_8_POE is a detergent that has been shown to specifically solubilize bacterial inner membranes.

### Production, Purification of the RNA Scaffold

Purification of the RNA scaffold, dubbed ref-f, was performed as described in Ponchon et al. (2013). Heterologous expression was achieved in XL1 cells. A pre-culture was grown 10 h at 37°C under shaking at 200 rpm and inoculated at OD_600nm_ = 0.05 in LB medium containing 100 μg/mL of ampicillin. Cell were grown overnight to OD_600nm_ = 3 at 37°C at 200 rpm. The expression of ref-f is induced by the addition of 1 mM of IPTG. After 3 h, cells were centrifuged at 7000 g, during 20 min at room temperature. Pellets were re-suspended in 1L of buffer containing 20 mM Tris, 200 mM NaCl and centrifuged at 7000 g, during 20 min, again at room temperature. Pellets were re-suspended in 40 mM of MgSO_4_, 50 mM Na_3_Citrate pH 5.6. RNA was extracted upon incubation with 40 mL of phenol during 2 h under gentle stirring at room temperature. The mixture was centrifuged 15 min at 3200 g. The supernatant was incubated with two volumes of ethanol and 1/20 of NaCl 5 M, a precipitate was formed and the mixture was centrifuged 5 min at 3200 g. The pellet was dried under the fume hood for 5 min and 10 mL of distilled water was added. The RNA was then purified by anion exchange chromatography.

### Preparation of Unilamellar Vesicles

A mix of lipids consisting of DOPC and DOPE with cap biotin at a 20:1 w/w ratio were dissolved in 25 mM HEPES pH7, 100 mM K_2_SO_4_ and 2 mM MgSO_4_, to a final concentration of 1 mg/mL. The suspension was then heated for 10 min at 37°C. This solution was then sonicated for 10 min with 30′ pulse/30′ pause cycles. Liposomes were subsequently passed through 200 nm membranes and then 100 nm membranes thanks to an extruder from Avanti (20 pass for each type of membrane). The homogeneity of the suspension was controlled by Dynamic Light scattering (DLS, Nanosizer Malvern).

### Reconstitution of the Proteins

Liposomes were first solubilized at a 1:1 detergent to lipid ratio (w/w). Solubilization was performed using ß-OG for 1 h at 20°C under gentle agitation. Proteins were added to the solubilized liposome suspension at the following lipid-to-protein ratio (w/w): MexB 1:20; MexA, 1:30; OprM, 1:20. OprM proteoliposomes were prepared in the presence of ref-f at 3 mg/ml: MexAB proteoliposomes were prepared the presence of 3 mM pyranine. Detergent removal was achieved upon 3 consecutive additions of Bio-beads (previously washed with methanol, ethanol and water) at a Bio-bead: detergent ratio of 20 (w/w) for at 1 h at 20°C, followed by an eventual addition, overnight, at the same ratio. MexAB proteoliposomes were purified from untrapped pyranine using a PD-10 desalting column (GE Healthcare). OprM proteoliposomes were purified from untrapped RNA using source Q column (GE Healthcare).

The efficiency of the reconstitution is controlled by running a discontinuous sucrose gradient formed of five layers (60, 20, 10, 5 and 2.5% sucrose). After ultracentrifugation of liposomes on this sucrose gradient overnight at 100,000 g, non-incorporated proteins are found at the bottom of the tube, while proteoliposomes are trapped at a sucrose interface that corresponds to their intrinsic density. Empty liposomes are recovered higher in the gradient. Different gradient fractions are collected gently and analyzed on SDS-PAGE using Coomassie staining.

### Ethidium Bromide Transport

Ethidium bromide (EthB) transport was measured as previously described (Verchère et al., 2015). Fluorescence measurements were conducted at 25°C using a SAFAS-Xenius spectrofluorimeter. The measurements were performed using the dual-wavelength mode, with excitation and emission wavelengths set at 300 nm and 600 nm, respectively, for the recording of EthB fluorescence and at 455 nm and 509 nm for the recording of pyranine fluorescence. Bandwidths were set at 10 nm. 100μL of OprM proteoliposomes and 100 μL of MexAB proteoliposomes were mixed and diluted with 800μL of the liposome reconstitution buffer (25 mM Hepes pH7, 100 mM K_2_SO_4_, 2 mM MgSO_4_). The system was then incubated until a steady baseline was obtained. EthB was then added at 5μM and a pH jump was performed after 20 min incubation.

### Pull Down Assay

100 μL of the respective liposomes were mixed and the suspension was then complemented with 800μL of the liposome reconstitution buffer (25 mM Hepes pH7, 100 mM K_2_SO_4_, 2 mM MgSO_4_), when the assembly is performed under non-energized conditions (see § Investigation of the assembly) or with 800μL of a buffer containing 20 mM Mes Tris pH6, 100 mM K_2_SO_4_, 2 mM MgSO_4_ when the assembly is performed under pH gradient conditions (see § Liposome pull down as a complementary approach to a transport assay, below). Both buffers have been tuned in order to have the same osmolarity, as measured by a cryoscopic Osmometer (Fisher Scientific, reference: 11900557). Then, the liposomes are incubated for 20 min at room temperature. Finally, 5 μL of MagStrep resin (MagStrep “type2HC” high capacity beads, IBA) at 5% (50 mg/mL) was added and the suspension was then placed on a magnetic separator (IBA, ref. 2-1602-000) in order to magnetize down the MexAB-biotin enriched-proteoliposomes and their potential interacting partner. Within a couple of minutes, the target proteins are specifically pelleted. The corresponding pellets were washed twice with 1 ml of 100 mM Tris HCl pH8, 150 mM NaCl before being resuspended directly in a 5X acrylamide gel loading buffer. The proteins were revealed on 10% gels by Laemmli-type SDS-PAGE (Laemmli, 1970) and stained with Coomassie blue.

## Results

### Investigation of the Assembly

**FIGURE 1:**
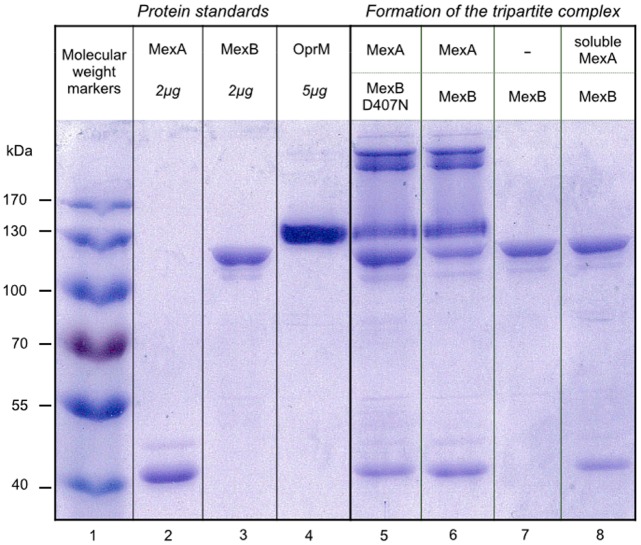
**MagStrep-mediated pulldown of proteoliposomes allows for an identification of the formation tripartite complexes.** Lane 1 contains molecular mass markers (PageRuler Prestained Protein Ladder from Thermo Scientific, #26616). Detergent-purified MexA, MexB, OprM are loaded (2–5 μg protein per lane) onto a 10% SDS-PAGE Laemmli gel as reference (lanes 2, 3, and 4, respectively) and stained with Coomassie Blue. Proteoliposomes containing MexA and MexB (lane 5), MexA and MexB_D407N_ (lane 6), MexB alone (lane 7), or MexB in the presence of a soluble version of MexA (lane 8) were mixed with proteoliposomes containing OprM as described in the Material and Methods section. Liposomes were sedimented and the pellets were mixed with 5 μL loading buffer before being loaded on the gel. The samples were not boiled in order to avoid aggregation of the membrane proteins.

Streptavidin- or streptactin-coated beads are classically used to purify biotinylated proteins, or proteins containing a strep tag, by affinity chromatography procedures. Here, we take advantage of streptavidin-coated magnetic beads to specifically sediment MexAB proteoliposomes containing biotinylated lipids. As can be seen Figure 1, when MexAB proteoliposomes are preincubated with OprM proteoliposomes, OprM is eventually found in the pellet after sedimentation by the streptavidin-coated magnetic beads, thereby providing a clear evidence of the assembly *in vitro* (see the presence of MexA, MexB, and OprM, lane 6, by comparison with the protein standards lane 2, 3, and 4, respectively). Note that in addition to the presence of OprM itself, one can see complexes of very high molecular mass in the upper part of the gel. These complexes were extracted from the gel, incubated for 1.5 h in water at room temperature and treated under mild heating conditions (60°C for 15 min in SDS PAGE loading dye). This revealed that the high molecular mass complexes were OprM aggregates (data no shown). As a control, we have performed the same experiment in the absence of MexA. MexA is a periplasmic protein that is covalently attached to the membrane via a lipid anchor. When MexB is reconstituted in the absence of MexA, OprM is not found in the pellet (lane 7), in accordance with the well-documented role of MexA on the formation of the pump. Interestingly, when a soluble version of MexA is added on the latter system, the formation of the complex is not restored (lane 8). Once again, this result is reminiscent with studies that showed that the absence of the lipid anchor severely impairs the ability for the efflux pump to form (Ferrandez et al., 2012). It must be put forward that the soluble version of MexA does not restore the ability to form the complex but is nevertheless able to bind to MexB, as both MexA and MexB are identified on lane 8. Finally, we also decided to study if the complex can form if the experiment is performed in the presence of an inactive version of MexB, mutated in the proton relay pathway (D407N mutant, Guan and Nakae, 2001; Su et al., 2006; Takatsuka and Nikaido, 2006), and in the presence of the palmitoylated version of MexA. Lane 5 shows that the complex does form in this condition showing that the mutation, located in the transmembrane part of the protein, does not affect the formation of the pump.

### Liposome Pull Down as a Complementary Approach to a Transport Assay

**FIGURE 2:**
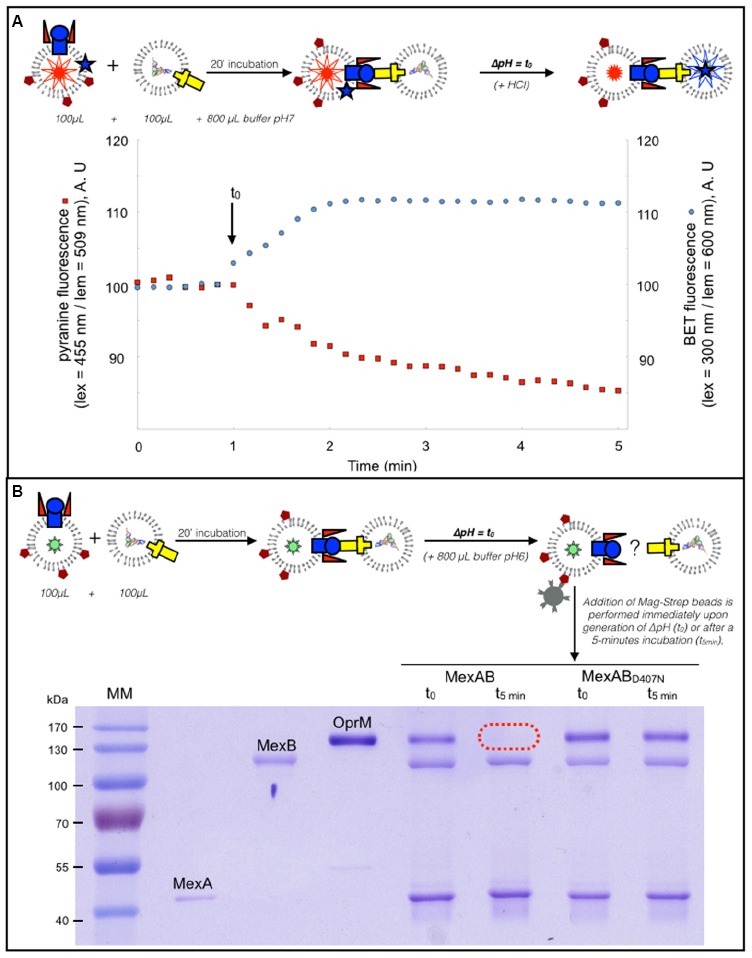
**The pull down assay can be adapted in conditions mimicking the transport. (A)** Dual wavelength fluorescence monitoring of transport. Fluorescence measurements were conducted with excitation and emission wavelengths set for the recording of EthB and pyranine fluorescence. OprM proteoliposomes and of MexAB proteoliposomes were mixed and incubated with EthB (5 μM) and a pH jump, indicated by an arrow, was performed after 20 min incubation. The concomitant fluorescence variations of pyranine (see **A**, red trace) and EthB (see **A**, blue trace) is the proof of actual transport of the substrate from one vesicle to the other. **(B)** MagStrep-mediated pulldown of proteoliposomes under conditions of a pH gradient. The liposomes used in the experiment shown **(A)** were enriched with biotinylated lipids in order to investigate the kinetics of the tripartite complex assembly in the context of transport. Samples were prepared as described in the Material and Methods section and were incubated for 20 min before being diluted with a buffer containing 20 mM Mes Tris pH6, 100 mM K_2_SO_4_, 2 mM MgSO_4_ in order to generate a pH gradient. Streptavidin-coated magnetic beads were then added either immediately (t_0_) or after a 5-min incubation (*t* = 5 min).

We have previously shown that transport through the whole tripartite pump can be measured thanks to the use proteoliposomes loaded with fluorescent probes that report the activity of MexB and OprM (Verchère et al., 2015). In brief, MexA and MexB are reconstituted in proteoliposomes containing pyranine, while OprM is reconstituted in another kind of proteoliposome in which RNA is encapsulated. Besides, EthB, a substrate of the pump that has DNA-intercalating properties is incubated with the proteins. Pyranine is a pH-dependent probe which fluorescence decreases when pH decreases. EthB fluorescence increases significantly upon intercalation into nucleic acids. Because the pump is energized by the proton motive force (Thanassi et al., 1997; Li et al., 1998; Zgurskaya and Nikaido, 1999), transport can be triggered by generating a change in the pH in the external medium (the pH jump is indicated by an arrow, Figure 2A). Efflux is monitored by measuring the simultaneous fluorescence variations of pyranine (see Figure 2A, red trace) and EthB (see Figure 2A, blue trace): the concomitant fluorescence variations indicate actual transport of the substrate from one vesicle to the other (the increase of EthB fluorescence is ascribed to the intercalation into RNA), mediated by an active transport through the pump (the decrease of pyranine fluorescence is the result of proton counter-transport, hence the acidification of the liposome, by MexB). If the same experiment is now performed in the presence of proteoliposomes where the MexAB_D407N_ inactive mutant is used, both the pyranine and EthB fluorescence signals remain steady (see Verchère et al., 2015). The liposomes used in the experiment shown Figure 2A were enriched with biotinylated lipids in order to investigate the kinetics of the tripartite complex assembly in the context of transport, using the pull-down protocol on the very same proteoliposome preparation. To that purpose the proteoliposomes were incubated as described in the previous paragraph except that the buffer used to dilute the liposomes was now more acidic than that in which the proteoliposomes were prepared. As a consequence, a pH gradient is generated across the liposome membrane. As can been seen Figure 2B, the tripartite complex is observed upon generation of the pH gradient, as OprM is detected together with MexA and MexB (see “MexAB samples” prepared at t_0_). However, after transport has reached steady state (i.e., within a couple of minutes), the complex has seemingly disappeared. Indeed, OprM does no longer interact with MexAB (see the “MexAB samples” prepared at *t* = 5 min). Interestingly, when the same experiment is performed in the presence of the D407N mutant, the complex remains stable (see “MexAB_D407N_ samples” prepared at t_0_ and at *t* = 5 min).

## Discussion

### An Original Technique Allowing to Monitor Transport and Assembly

The formation of the macromolecular complex formed by MexA, MexB, and OprM has been studied for long, by use of various biochemical and biophysical techniques. Theses studies made it possible to understand key aspects of the mechanism of an efflux pump but they are rather demanding in term of protein quantity (ITC) and methodology (in SPR, the target protein must be attached on the sensor chip and must remain stable throughout the whole titration experiment). As presented here, our protocol is much more versatile, it is compatible with a medium-large screening of potential inhibitors as it does not require large amounts of protein (typically 10 μg of protein is necessary for one measurement). Finally, this protocol can be scaled up in 96-wells. In such a case, SDS-PAGE would not be the best identification option but we have adapted the protocol by using OprM proteoliposomes enriched with lipids grafted with a rhodamine function. In such a context, the purple color of the liposomes remains in the supernatant when the complex is not formed, the purple color being lost in the pellet if the complex forms (data not shown). Hence, the assembly could be simply monitored by colorimetry. Our methodology finds its limit in being only qualitative. Indeed, the variability of the efficiency of reconstitution, and the reproducibility of the procedure (washing of the pellets, resuspension in the loading buffer) precludes from any quantitative conclusion. Hence, we can not claim any information on the stoichiometry.

### Role of MexA

The results presented Figure 1 show that MexA is absolutely necessary for the assembly, a result in accordance with previous results. Tikhonova et al. (2011) showed that if an His-tag is located on AcrA or AcrB, a tripartite complex can be purified in measurable quantities from bacterial native membranes. However, if the His-tag is placed on TolC, only traces of the complex are purified. AcrA is thus indispensable for AcrB to co-fractionate with OprM (Tikhonova and Zgurskaya, 2004). The interaction between the different partners has also been investigated using ITC, which allows evaluating the affinity between two components (Touzé et al., 2004). When the interaction between AcrB and TolC is investigated by ITC measurement, no stable interaction is detected. This was interpreted by the fact that AcrA is required for the interaction between AcrA and TolC.

Finally, the efflux pump MtrC-MtrD-MtrE (respectively MFP-RND-OMF) from *Neisseria gonorrhoea* has also been intensely investigated *in vivo* by Janganan et al. (2013). They made use of vancomycin susceptibility to monitor the assembly of the OMF with its RND and MFP partners (Janganan et al., 2013). Gram negative bacteria are not susceptible to vancomycin but become so if the OMF were to open and let the antibiotic diffuse inside the cell. Interestingly if the MFP is expressed together with the OMF, cells become sensitive to vancomycin, meaning that the MFP is required for the OMF to open. Note that, it was shown that a truncated version of the MFP, consisting of the alpha helical hairpin domain only, is not able to open the OMF. Finally the assembly of the whole complex was monitored by SPR (Tikhonova et al., 2011), showing that AcrA-AcrB and AcrA-TolC complexes can be detected. If palmitoylated AcrA and TolC are premixed and then injected on AcrB adsorbed on solid support, the complex does not form, probably because TolC inhibits the interaction between AcrB and AcrA.

The reason for the fact that the soluble MexA does not restore the formation of the complex remains an open question. It could very well be that this construction is intrinsically unable to make the pump assemble but one can also imagine that this inability to form a complex is purely thermodynamic. Indeed, the lipid anchor may be present for restricting the diffusion of the protein to a two-dimensional space, thereby allowing a more probable interaction with MexB. However, the role of the N-terminal lipid part of AcrA, was investigated in real time by SPR (Tikhonova et al., 2011) and it was shown that when the MFP lacks its lipid anchor, no oligomerization of the MFP is detected. Considering that AcrA binds AcrB as a dimer, it seems probable that the lipid anchor has a genuine effect on the assembly. We have to stress that the observation that the soluble version of MexA is nevertheless able to bind MexB (see Figure 1, lane 8) is in contradiction with the fact that Tikhonova et al. showed that the binding of soluble AcrA to AcrB is very weak at pH7.5 and pH6.

### Role of pmf on the Assembly

We show that the mutation within the proton relay pathway does not impair the assembly, which indicates that the efflux pump assembly does not require transport activity. Again, this result is in line with the work of Tikhonova and Zgurskaya regarding the assembly of the AcrAB-TolC pump from native bacterial membranes (Tikhonova and Zgurskaya, 2004) where they showed that if the corresponding non-functional mutant of AcrB (AcrB Asp408Ala) is expressed, the complex is still co-purified. Besides, if the proton motive force is disrupted in the cells (using CCCP, valinomycin and/or nigericin) the complex is also co-purified.

### Role of pmf on the Disassembly

In a remarkable series of papers, Bavro et al. (2008) previously investigated the energy dependence of the pump assembly, by use of non- functional mutants of MtrD mutated in residues involved in the proton-transducing pathway (Asp405, Asp406, and Lys948). If these mutants are expressed with the MFP and the OMF, cells remain insensitive to vancomycin; indicating that the efflux pump must dissociate under the proton motive force. Although very elegant, this conclusion is indirect and is realized in the context of the bacteria where additional factors could be suspected. Figure 2 provides direct and non-ambiguous support for the fact that the proton motive force indeed promotes disassembly of the complex, as evidenced by i) the disappearing of OprM when the wild type pump has reached its steady state and ii) by the maintaining of the assembly when the D407N mutant, defective in proton translocation, is used.

## Conclusion

We were able to detect the assembly of an efflux pump after it has been reconstituted into proteoliposomes. We could discriminate between conditions that allow, or favor, the formation of the complex and could also confirm that MexA is mandatory for the assembly to take place. Hence, this is in accordance with several studies obtained in recent years on that matter. Our reconstitution method and the subsequent characterization approach described in the present work allow us to consider further studies of reconstitutions including proteins mutated at various positions, in order to shed light on the actual determinants of transport in a simple system in a bottom-up approach.

## Author Contributions

Conceived and designed the experiments: MP. Performed the experiments: YN, AV, MP. Wrote the paper: AV, GP, IB, MP.

### Conflict of Interest Statement

The authors declare that the research was conducted in the absence of any commercial or financial relationships that could be construed as a potential conflict of interest.

## Abbreviations

ßOG: beta-octyl glucopyranoside
DDM: dodecyl maltoside
EthB: Ethidium bromide.
DOPC: 1,2-dioleoyl-sn-glycero-3-phosphocholine
DOPE: 1,2-dioleoyl-*sn*-glycero-3-phosphoethanolamine
RND: Resistance, Nodulation, cell Division family
OMF: outer membrane factor family
MFP: Membrane Fusion family
ITC: isothermal titration calorimetry
SPR: surface plasmon resonance.
